# Epidermal Growth Factor Receptor Tyrosine Kinase Inhibitors in Cancer: Current Use and Future Prospects

**DOI:** 10.3390/ijms251810008

**Published:** 2024-09-17

**Authors:** Henry Dickerson, Ahmad Diab, Othman Al Musaimi

**Affiliations:** 1 School of Pharmacy, Newcastle University, Newcastle upon Tyne NE1 7RU, UK; h.dickerson2@newcastle.ac.uk (H.D.); a.diab2@newcastle.ac.uk (A.D.); 2 Department of Chemical Engineering, Imperial College London, London SW7 2AZ, UK

**Keywords:** tyrosine kinase inhibitors, epidermal growth factor receptors, NSCLC, gefitinib, afatinib

## Abstract

Tyrosine kinase inhibitors (TKIs) have emerged as a leading targeted cancer therapy, reducing the side effects often seen with non-targeted treatments, especially the damage to healthy cells. To tackle resistance, typically caused by epidermal growth factor receptor (EGFR) mutations, four generations of TKIs have been developed. Each generation has shown improved effectiveness and fewer side effects, resulting in better patient outcomes. For example, patients on gefitinib, a first-generation TKI, experienced a progression-free survival (PFS) of 10 months compared to 5 months with conventional chemotherapy. Second-generation TKI afatinib outperformed erlotinib and extended PFS to 11.1 months compared to 6.9 months with cisplatin. Third-generation TKIs further increased survival to 38.6 months, compared to 31.8 months with first-generation TKIs. This progress demonstrates the ability of newer TKIs to overcome resistance, particularly the T790M mutation, while reducing adverse effects. Ongoing research focuses on overcoming resistance from newer mutations like C797S to further improve patient survival. These developments highlight the significant progress in TKI therapy and the continued effort to refine cancer treatment. Recent research in South Korea shows that third-generation TKIs are ineffective against non-small cell lung cancer (NSCLC) with the C797S mutation. Several trials have started showing promising in vitro and in vivo results, but more trials are needed before clinical approval. This review underscores notable advancements in the field of EGFR TKIs, offering a comprehensive analysis of their mechanisms of action and the progression of various TKI generations in response to resistance.

## 1. Introduction

### 1.1. Cancer

The National Health Service (NHS) in the UK defines cancer as a condition where cells grow and reproduce uncontrollably, with the potential to spread to other body parts through metastasis. The most common cancers in the UK are breast, lung, prostate, and bowel cancer. Early symptoms often include a lump in the affected area. Diagnosis usually involves a combination of screening tests, urinalysis, and blood analysis, as no single test can definitively diagnose cancer [1,2].

In 2012, cancer caused a loss of 281,373 life years, with an average of 19.64 years per person. Productivity losses totaled USD 149 million for males and USD 121 million for females, with the top five cancers responsible for nearly half of the burden in males and over 60% in females [3]. In 2018, costs were GBP 18.9 billion for cancer, GBP 12.7 billion for coronary heart disease, GBP 11.7 billion for dementia, and GBP 8.6 billion for stroke [4]. By 2050, costs are projected to rise to GBP 26.5 billion, GBP 19.6 billion, GBP 23.5 billion, and GBP 16.0 billion, respectively [4]. A study by Chen and colleagues estimated the global economic cost of cancers from 2020 to 2050 at USD 25.2 trillion, with unequal distribution of economic and health burdens across countries, regions, and income groups [5].

### 1.2. Treatment

#### 1.2.1. Non-TKI Agents

In the UK, it was estimated that 3 million people were suffering from cancer as of October 2022, and this number is expected to rise to 5.3 million by 2040 [6]. On average, in 2019, a new person was diagnosed with cancer every 90 s, and there were 460 cancer-related mortalities per day [6]. Currently, post-diagnosis cancer treatments include chemotherapy, radiotherapy, immunotherapy, molecular targeted therapy, and surgery.

These treatments aim to inhibit cell proliferation and tumor multiplication, thereby preventing the growth of cancerous tumors. However, their selectivity is often poor, resulting in healthy cells being targeted and causing toxic side effects.

Cytotoxic drugs are categorized into classes such as alkylating agents [7], antimetabolites [8], hormone-based therapies [9], microtubule-targeting drugs [10], signaling pathway inhibitors [11], and enzyme inhibitors like histone deacetylase inhibitors (HDACs) [12]. In this review, we focus solely on alkylating agents because they were the only class of cytotoxic drugs compared against TKIs in the discussed clinical trials. Antibody–drug conjugates (ADCs) will be explored as a strategy for enhancing treatment specificity.

Alkylating agents like cisplatin inhibit DNA replication and transcription [13]. Cisplatin, a platinum coordination complex, enters cells via copper transporter 1 (CTR1) and becomes activated [14]. The replacement of chloride atoms by water molecules forms an electrophilic molecule that binds covalently to nucleic acids, leading to intra-strand and inter-strand crosslinking of DNA, ultimately causing cancer cell apoptosis [15].

Despite its efficacy, cisplatin’s non-selectivity results in significant toxicity to non-cancerous cells, especially affecting the heart, kidneys, and liver in 25% to 36% of patients [15]. Cisplatin, first synthesized in 1844, became the first platinum-based chemotherapy agent approved by the U.S. Food and Drug Administration (FDA) in 1978. Its second-generation counterpart, carboplatin, aimed to reduce toxicity by lowering the number of non-cancerous cells affected during treatment. Carboplatin’s slower hydration rate and cyclobutane dicarboxylic acid ligands result in reduced hepatotoxicity, nephrotoxicity, neurotoxicity, and ototoxicity compared to cisplatin [16]. Approved in 1986, carboplatin is used in high-dose chemotherapy for aggressive tumors but remains prone to drug resistance, a persistent issue in platinum-based treatments.

However, treatments like cisplatin and carboplatin can significantly affect patients’ quality of life, leading to more time away from work, extended hospital stays, and an increased burden of mental health issues such as anxiety and distress. These challenges contribute to poor medication adherence, complicating the treatment process for cancer patients [17].

**Figure 1:**
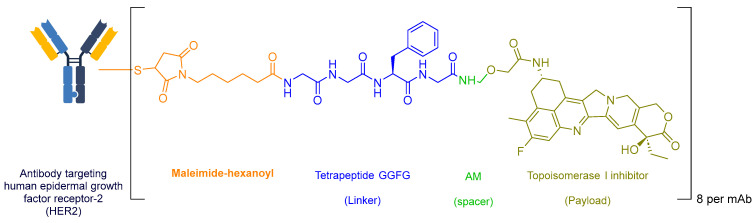
Chemical structure of Enhertu.

Enhancing the targeting efficiency of oncology treatments is a key strategy for increasing their specificity. ADCs are crucial in addressing the limitations of untargeted cancer therapies. ADCs consist of a drug, such as a chemotherapeutic agent, linked to a monoclonal antibody (mAb) through a cleavable or non-cleavable linker. This design allows the ADC to specifically bind to receptors that are overexpressed in cancer cells. Once bound, the ADC is internalized, and the drug payload is delivered to the therapeutic target [18]. An example of an ADC is fam-trastuzumab deruxtecan-nxki (Enhertu) (Figure 1) [19].

Enhertu combines a topoisomerase inhibitor payload with a human epidermal growth factor receptor 2 (HER2)-directed antibody to target HER2, a protein that is overexpressed in certain cancers. The ADC features a Gly-Gly-Phe-Gly tetrapeptide linker that connects the HER2 antibody to the drug. When Enhertu binds to the HER2 receptor on cancer cells, it is internalized, and the linker is cleaved by lysosomal enzymes, releasing the drug inside the cell [19]. The topoisomerase inhibitor then induces DNA damage by preventing the topoisomerase enzyme from unwinding DNA strands during replication, specifically in malignant cells, leading to cell apoptosis and a reduction in tumor size. Enhertu was developed by Daiichi Sankyo and received FDA approval in 2019 for the treatment of metastatic NSCLC with a HER2 mutation [19].

#### 1.2.2. TKI Agents

TK mutations contribute significantly to cancer development, primarily through gain-of-function mutations that hyperactivate downstream signaling pathways. These mutations circumvent normal physiological controls, promoting uncontrolled cell growth and proliferation. An important concept in this context is the identification of “driver mutations,” which are mutations that provide cancer cells with a selective growth advantage. Understanding these mutations is crucial for unraveling the initiation and progression of cancer and offers opportunities for developing targeted therapies.

A key area of focus is the EGFR, which is commonly affected by somatic mutations within its TK domain. These mutations, especially those affecting the DGF motif and nucleotide-binding pocket within the N-lobe of the intracellular region of EGFR, disrupt adenosine triphosphate (ATP) binding and enhance catalytic activity [20]. This hyperactivation leads to the oncogenic properties observed in various cancers, such as glioblastoma and non-small cell lung cancer (NSCLC) [21]. EGFR mutations have been identified in various domains of the protein, including the extracellular, transmembrane, and juxtamembrane regions, each contributing to tumorigenesis in distinct ways. For example, extracellular mutations in the EGFR have been linked to glioblastoma and are often associated with EGFR overexpression [22]. However, these mutations render TKIs ineffective, necessitating the exploration of alternative therapies.

TKIs were developed to address the limitations of non-specific cancer therapies, such as alkylating agents like cisplatin, which target both healthy and cancerous cells, leading to severe toxicity. Unlike traditional chemotherapy, TKIs specifically target cancer cells by inhibiting autophosphorylation at the EGFR, reducing toxicity and improving patient outcomes. Moreover, TKIs are more convenient, as they can be administered orally, a significant advantage over intravenous chemotherapy [23]. Studies have shown that the majority of cancer patients prefer oral treatments over intravenous options, with 84.6% favoring the former due to their convenience and reduced hospital visits [24].

**Figure A1:**
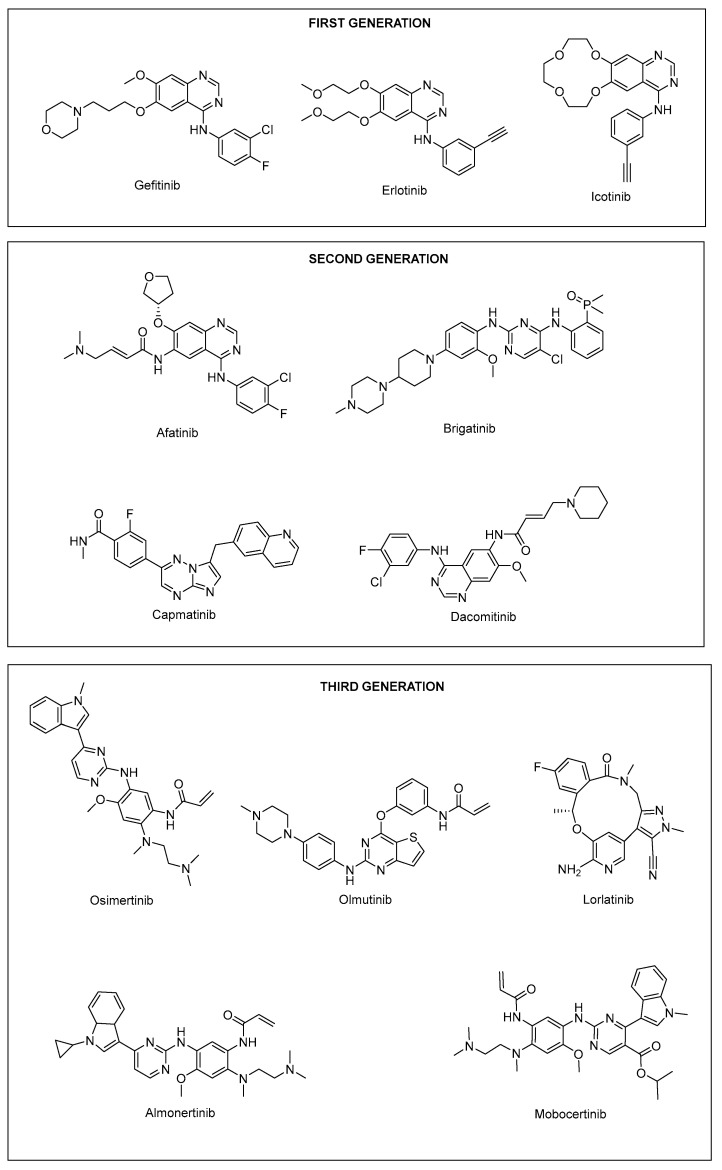
Chemical structures of all 12 EGFR TKIs used to treat NSCLC, separated into respective generations.

As of 2021, 12 EGFR-TKIs have been approved for the treatment of NSCLC, classified into first-, second-, and third-generation TKIs. These drugs are licensed for clinical use worldwide by major regulatory agencies, including the FDA, European Medicines Agency (EMA), and authorities in Japan, China, and South Korea. This review delves into the efficacy and benefits of TKIs, underscoring their role in modern cancer treatment, particularly for NSCLC, which accounts for 85% of all lung cancer cases (Table 1) (Figure A1) [25,26].

## 2. EGFRs

### 2.1. Structure and Function

EGFRs are widely expressed across various cell types in the body, including vascular endothelial cells, HeLa cells, conjunctiva cells, uterine smooth muscle cells, keratinocytes, amniotic cells, and placental membranes [38]. While in normal tissues the receptor count typically does not exceed 100,000, in cancerous cells, EGFR expression can skyrocket to as many as 2 million receptors [38]. This massive overexpression is a key factor in promoting uncontrolled cell proliferation, inhibiting apoptosis, and facilitating tumor growth.

**Figure 2:**
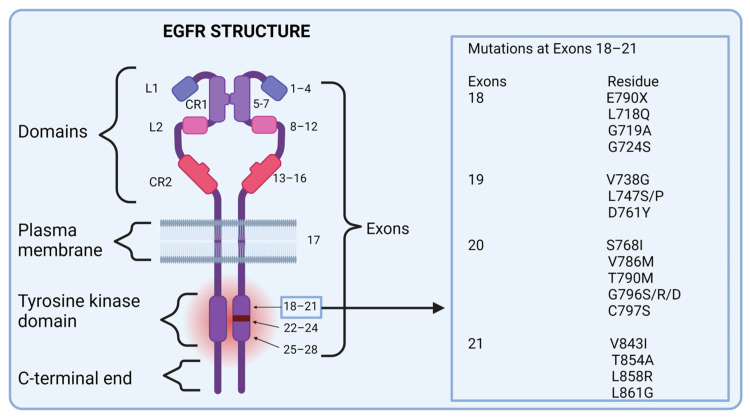
EGFR structure, including domains, exons, and respective mutations.

The critical role of the EGFR in cancer development was discovered in the 1960s by Stanley Cohen, who established a link between elevated EGFR levels and malignancy [39]. The EGFR belongs to the erythroblastic oncogene B (ERBB) family of receptors and is a transmembrane protein with an intracellular kinase domain. The receptor’s structure allows it to bind extracellular protein ligands, which in turn triggers a cascade of intracellular signaling promoting cell growth and survival. The EGFR itself is a 170 kDa molecule consisting of a single polypeptide chain that spans 1186 amino acids and contains 28 exons (Figure 2) [40].

This discovery paved the way for targeted cancer therapies aimed at inhibiting EGFR signaling, leading to the development of TKIs, which block the receptor’s activity and reduce cancer cell proliferation.

The EGFR is composed of three primary regions: the extracellular, transmembrane, and intracellular regions, each playing crucial roles in its function. (i) Extracellular region: this region includes exons 1–16 and is responsible for regulating the binding of ligands to the EGFR molecule. When ligands such as EGF bind to this region, it triggers the receptor’s activation. (ii) Transmembrane region: this region is composed of exon 17, which anchors the receptor into the cell membrane. It is a single hydrophobic region that facilitates the receptor’s positioning and stability within the membrane. (iii) Intracellular region: comprising exons 18–28, the intracellular region contains the TK domain, which is crucial for receptor autophosphorylation. This process is essential for activating downstream signaling pathways that control cell proliferation, survival, and other cellular functions [39].

The TK domain is susceptible to mutations, particularly in exons 18–21, which are associated with resistance to TKIs. The most common mutations, occurring in 85–90% of cases, include exon 19 deletions and the L858R point mutation in exon 21. Less common mutations (10–15%) involve mutations in exon 18 (point mutations), exon 21 (L861X), and exon 20 (insertions and S768I) [41].

The intracellular TK domain also contains several functional regions: (i) the N-lobe, which includes the nucleotide-binding site, P-loop, and αC-helix, (ii) the C-lobe, which contains the DFG motif (critical for activation), (iii) the catalytic loop, (iv) and the highly flexible activation loop (A-loop) [39]. These regions help regulate the receptor’s ability to bind ATP and undergo the conformational changes necessary for kinase activation. When ligands bind to the extracellular region, the receptor undergoes a conformational change, promoting homo- or heterodimerization (pairing with itself or other receptors), which is necessary for signal transduction [40].

Understanding these regions and their mutations is key in the development of targeted therapies such as TKIs, aimed at inhibiting abnormal EGFR activity and preventing uncontrolled cell proliferation.

### 2.2. Physiological Activation

The physiological activation of the EGFR and the subsequent transmission of its signal within the cell is a multi-step process that triggers critical intracellular pathways, which are often hijacked in cancerous cells, particularly in non-small cell lung cancer (NSCLC). (i) Ligand binding and dimerization: the process begins when an extracellular ligand, such as EGF, binds to the extracellular domain of the EGFR monomers. This ligand binding induces a conformational change, leading to the dimerization of the EGFR molecules, forming either homodimers (EGFR-EGFR) or heterodimers (EGFR paired with another ERBB receptor). (ii) TK activation: dimerization results in the activation of the intrinsic TK within the intracellular domain of the EGFR. The TK then autophosphorylates several tyrosine residues on the receptor’s intracellular tail. These phosphorylated residues act as docking sites for downstream signaling proteins. (iii) Activation of rat sarcoma (RAS): one of the key proteins activated by these phosphorylated tyrosine residues is the RAS protein. Once activated, RAS acts as a molecular switch that further transmits the signal downstream.

### 2.3. Signaling Pathways

**Figure 3:**
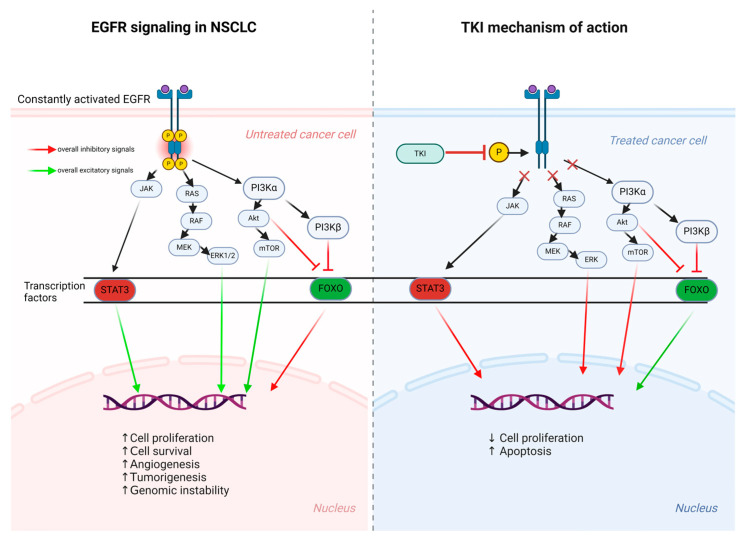
Molecular signaling of the EGFR and the resulting biological response, under pathological and treatment states [42].

This pathway is important for cell survival and growth. In NSCLC, elevated PI3K levels, triggered by phosphorylated TK, activate AKT, which promotes cell survival and proliferation. Disruption of this pathway is linked to tumor formation in NSCLC (Figure 3) [42]. Mitogen-activated protein kinase (MAPK) pathway: RAS binds to phosphorylated kinase residues, increasing MAPK levels. This pathway is crucial for cell division and growth [43].

### 2.4. The Forkhead Box FOXO Regulation

The FOXO family of transcription factors typically induces apoptosis and regulates cell cycle arrest. In NSCLC, FOXO is underregulated, leading to uncontrolled cell survival and proliferation, which helps cancer cells persist [42].

### 2.5. Additional Oncogenic Signaling

Key proteins involved in oncogenic signaling include ERK1/2 and STAT, which are excessively activated and promote tumor cell proliferation and survival. Elevated ERK1/2 and STAT, combined with underregulated FOXO, contribute to the continuous growth and spread of tumor cells [42].

In summary, EGFR activation triggers a network of signaling pathways, including PI3K-AKT and MAPK, which drive cancer cell survival and proliferation when dysregulated. Understanding these pathways is key to developing targeted therapies for cancer treatment.

## 3. TKI Development

Due to the EGFR’s pivotal role in cancer pathology, EGFR inhibitors have become a promising avenue for therapeutic intervention. Building on Stanley Cohen’s discoveries in the 1960s regarding the EGFR’s connection to malignancy, Dr. John Mendelsohn expanded this research in the 1980s and proposed that blocking EGFR signaling could serve as an effective anti-cancer treatment [44].

In 1983, Mendelsohn’s team developed two murine monoclonal antibodies (mAbs 225 and 528) specifically designed to target and inhibit the EGFR [44]. These antibodies blocked the TK activity within the EGFR, effectively preventing the phosphorylation process that triggers downstream pathways, such as the PI3K pathway. By inhibiting this pathway, these antibodies were shown to reduce cell proliferation and increase apoptosis in cancer cells [44]. This groundbreaking discovery laid the foundation for the development of EGFR-targeted therapies and sparked the creation of the first-generation TKI, gefitinib.

Since then, continued research has led to the development of second-, third-, and fourth-generation TKIs, each designed to address resistance mechanisms and improve patient outcomes. These advancements in TKI therapy have revolutionized the treatment of EGFR-driven cancers, particularly NSCLC, providing more targeted, effective, and personalized treatment options.

### 3.1. First-Generation TKI

**Figure A2:**
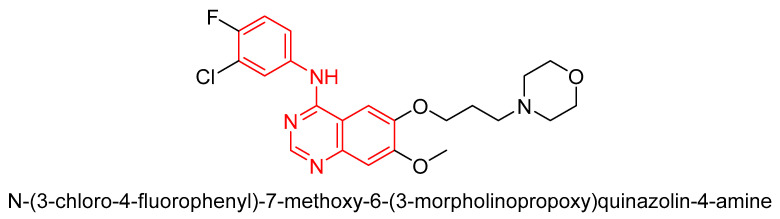
Chemical structure of gefitinib; red, anilinoquinazoline core.

Gefitinib, the first discovered TKI, is classified as a small molecule within the anilinoquinazoline class [45]. This structure is central to gefitinib’s function as an EGFR inhibitor, specifically targeting the TK domain of the receptor (Figure A2).

Gefitinib demonstrated significant inhibitory potency in vitro, with an IC50 value of 0.033 µM against the EGFR [46]. In clinical studies, gefitinib exhibited high and sustained blood levels over a 24 h period, with a bioavailability of 60% [47]. This compound also demonstrated high selectivity towards enzymes within the EGFR family, contributing to its effectiveness as a targeted cancer therapy.

**Figure 4:**
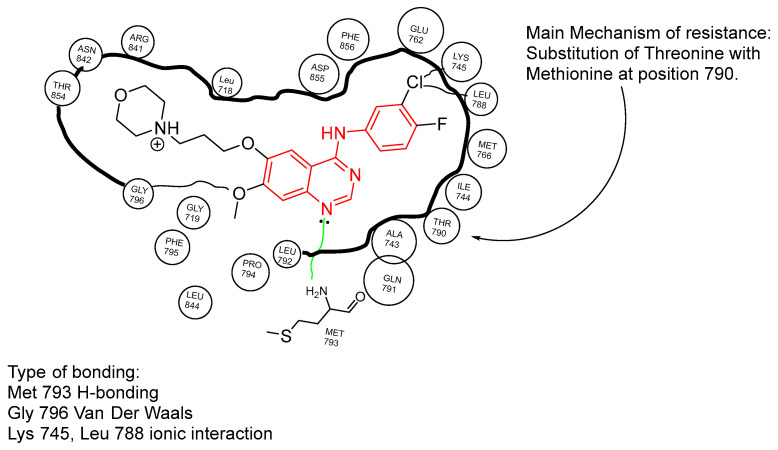
Schematic representation of gefitinib inside the EGFR-TK pocket.

The efficacy of gefitinib stems from its 4-anilinoquinazoline core, which serves as a potent EGFR-TK inhibitor by mimicking ATP and preventing the phosphorylation of EGFR. Specifically, gefitinib binds to the T790 residue of the EGFR through a hydrogen bond formed between the nitrogen on the 4-anilinoquinazoline core and Met793. Additionally, it interacts with the L858 residue via multiple carbon–hydrogen bonds with Leu718, Gln791, Pro794, and Gly796, contributing to its robust inhibition of EGFR signaling (Figure 4).

When analyzing the structure–activity relationship (SAR) of gefitinib, the 4-anilinoquinazoline core is pivotal for its interaction with the EGFR. This core facilitates hydrogen bonding with Met793, which is essential for the inhibitor’s binding and subsequent inhibition of the EGFR’s TK activity. However, gefitinib’s morpholine moiety is less effective in this regard; it lacks the necessary electron density and specific orientation to interact optimally within the TK pocket, which can lead to resistance in some cases [42].

In March 2006, AstraZeneca sponsored Phase III clinical trials to evaluate gefitinib’s efficacy and safety compared to traditional chemotherapy treatments. The study enrolled 230 patients with NSCLC from 43 institutions in Japan [48]. Patients were randomly assigned to receive either gefitinib at a daily dose of 250 mg administered orally or carboplatin administered intravenously over a 3 h period. The carboplatin dosage was individualized using the Calvert formula, which accounts for the patient’s creatinine clearance [48].

The trial, which concluded in December 2009, revealed that gefitinib significantly outperformed carboplatin in terms of PFS and overall survival (OS). The gefitinib group had a PFS of 10.8 months compared to 5.4 months for the carboplatin group. Additionally, the OS for the gefitinib group was 30.5 months with a 61.4% survival rate, compared to 23.6 months and 46.7% for the carboplatin group [48].

Despite these promising results, both treatment groups experienced adverse effects. For gefitinib, common side effects included diarrhea (*n* = 32), rash (*n* = 39), and elevated alanine aminotransferase (ALT) levels (*n* = 20). In contrast, carboplatin was associated with appetite loss (*n* = 39), sensory neuropathy (*n* = 28), arthralgia (*n* = 25), elevated ALT levels (*n* = 31), anemia (*n* = 35), and thrombocytopenia (*n* = 25) [48].

These findings underscore gefitinib’s efficacy as a targeted therapy with a more favorable safety profile compared to traditional chemotherapy, although both treatments carry specific side effects that need to be managed carefully (Table 2) [48].

The trial’s results indicate that gefitinib demonstrates superior efficacy and a more favorable side effect profile compared to carboplatin for treating NSCLC. The reduced toxicity of gefitinib makes it an attractive alternative to traditional chemotherapy, particularly for patients who are sensitive to the adverse effects of drugs like carboplatin.

However, several limitations of the study must be considered. The sample size of 230 patients, while adequate for initial findings, is relatively small compared to the large number of individuals affected by NSCLC. For instance, in 2023, approximately 238,340 adults in the United States were diagnosed with NSCLC [49]. A larger sample size might have highlighted statistically significant differences that were not apparent in this study.

Moreover, the trial’s results are based on Japanese patients aged under 75, without differentiation between genders. This narrow demographic focus raises concerns about the generalizability of the findings. Further research is needed to assess gefitinib’s efficacy and safety in older adults, who are more likely to suffer from NSCLC, and to understand its impact on smokers, another high-risk group for lung cancer [50].

Genetic differences between populations may also influence trial outcomes. For example, Japanese individuals have a distinct genetic profile compared to people in Western countries such as the US, UK, and Europe. Factors such as lower obesity rates, reduced saturated fat intake, and higher consumption of plant-based foods and non-sugar sweeteners contribute to lower cancer rates in Japan [51]. Consequently, findings from Japanese studies may not be directly applicable to Western populations, necessitating additional trials to evaluate the effectiveness of gefitinib in different genetic and lifestyle contexts.

**Figure 5:**
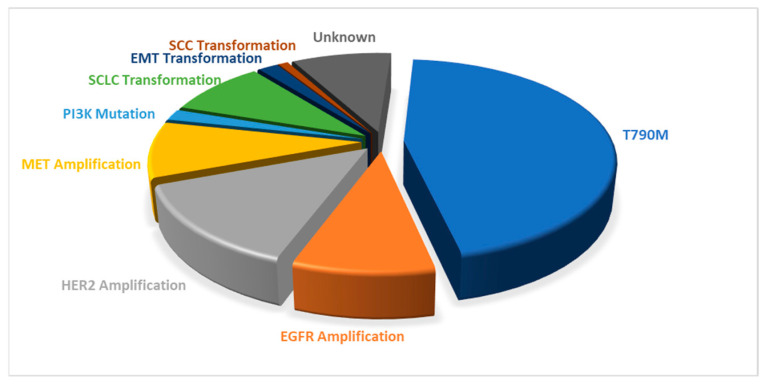
Resistance factors to first- and second-generation TKIs.

Gefitinib, approved by the FDA in 2003 and marketed as Iressa^®^, was the first generation of TKIs to gain clinical approval [52]. Despite its initial success, resistance to gefitinib developed due to the T790M mutation in exon 20 [53]. This mutation impairs the binding of gefitinib’s 4-anilinoquinazoline core to the T790 residue, thereby enabling EGFR to continue signaling and promoting tumor cell proliferation. Resistance typically emerges after 9–15 months of treatment, affecting approximately 50% of patients due to the T790M mutation (Figure 5) [39].

In conclusion, while gefitinib represents a significant advancement in targeted cancer therapy with notable efficacy and safety benefits over traditional treatments, ongoing research is crucial to address resistance mechanisms and validate its effectiveness across diverse patient populations.

### 3.2. Second-Generation TKI

To address the reduced efficacy of first-generation TKIs, particularly in the presence of the T790M mutation in the EGFR, second-generation TKIs were developed with structural enhancements to overcome this limitation. These second-generation TKIs, while maintaining the anilinoquinazoline core structure, incorporate additional features to enhance their effectiveness.

**Figure A3:**
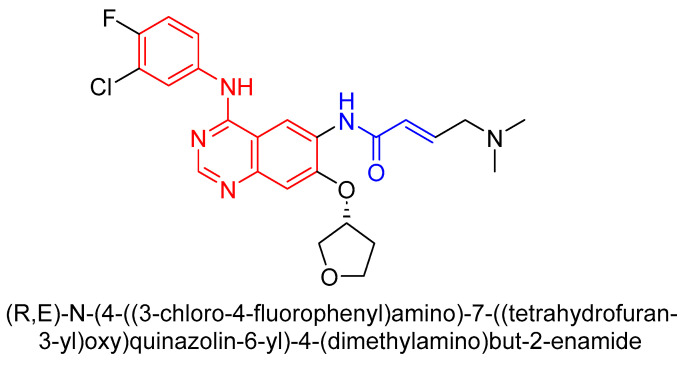
Chemical structure of afatinib. Red, anilinoquinazoline core; blue, covalent warhead.

Second-generation TKIs, such as afatinib, retain the anilinoquinazoline core that was crucial for the initial EGFR inhibition seen with first-generation TKIs [54]. However, they introduce a covalent warhead, which fundamentally changes their interaction with the EGFR (Figure A3). Specifically, afatinib includes an amide as its covalent warhead [54,55]. This modification enables afatinib to form an irreversible bond with the C797 residue on the EGFR. The irreversible nature of afatinib is a significant advancement, as it provides prolonged inhibition of the EGFR and overcomes resistance mechanisms associated with mutations like T790M.

#### 3.2.1. Structure and Mechanism of Second-Generation TKIs

Afatinib binds to the T790M mutation site through several interactions: (i) a hydrogen bond with Met793, a carbon–hydrogen bond with Gln791, and halogen interaction with Glu762 and L858 [56]. These additional interactions enhance afatinib’s potency compared to first-generation TKIs, although they may also lead to increased toxicity.

#### 3.2.2. Clinical Trials and Efficacy

Boehringer Ingelheim laboratories pioneered the synthesis of afatinib and subsequently conducted clinical trials to evaluate its efficacy and safety profile. These trials compared afatinib to first-generation TKIs like erlotinib and traditional chemotherapy agents such as cisplatin [57].

#### 3.2.3. Trial Comparison with Erlotinib (Table 3)

Erlotinib: A first-generation TKI with an anilinoquinazoline core that was effective but not irreversible. In trials, it served as a benchmark for evaluating afatinib’s enhanced efficacy and safety.

**Table 3:** Afatinib trial against erlotinib, including PFS, overall survival, objective response rate, disease control rate, as well as grade 3 adverse effects percentage [57].

Characteristics	Afatinib Group	Erlotinib Group
PFS	2.6 months	1.9 months
Overall survival	7.9 months	6.9 months
Objective response rate (%)	5.5	2.8
Disease control rate (%)	50.5	39.5
Grade 3 adverse effects (%)	57.1	57.5

#### 3.2.4. Trial Comparison with Cisplatin (Table 4)

Cisplatin: A traditional chemotherapy drug with known efficacy but significant side effects, which made it a relevant comparison for assessing the improved side effect profile of afatinib.

**Table 4:** Afatinib trial against cisplatin, including PFS, overall survival, tumor size change, and discontinuation rate due to adverse effects [57].

Characteristic	Afatinib Group	Cisplatin Group
PFS	11.1 months	6.9 months
Overall survival	33.3 months	21.1 months
Tumor size change (%)	−56.0	−23.0
Adverse effects discontinuation (%)	8.0	12.0

#### 3.2.5. Key Findings

Afatinib demonstrated superior efficacy in patients with NSCLC compared to both erlotinib and cisplatin. The irreversibility of afatinib’s binding to the EGFR allowed it to more effectively address mutations like T790M that contributed to resistance against first-generation TKIs. While afatinib showed increased potency, it also had a higher risk of toxicity compared to erlotinib. The introduction of the covalent warhead increased the drug’s interaction with off-target sites, which potentially led to more adverse effects.

In summary, second-generation TKIs like afatinib represent a significant advancement in targeted cancer therapy by overcoming resistance mechanisms associated with the T790M mutation and offering more potent inhibition of the EGFR. However, their increased potency comes with a trade-off in terms of potential toxicity, necessitating careful management of side effects in clinical practice.

This separate trial comparing afatinib with cisplatin highlights a clear benefit for afatinib, not only in terms of reducing tumor size but also in having a lower side effect profile compared to traditional chemotherapy. This encourages patients to continue their anticancer treatment with TKIs. Additionally, the trial results from AstraZeneca and Boehringer Ingelheim reinforce the idea that TKIs, both first- and second-generation, are increasingly successful in replacing traditional therapies like cisplatin for first-line treatment of NSCLC. This underscores the need for continued investment and development in this area of oncology.

Boehringer Ingelheim also sponsored Phase II trials that compared afatinib with gefitinib. Conducted between December 2011 and August 2013 across 64 centers in 13 countries, these trials involved 219 patients with NSCLC who were randomly assigned to the treatments [58]. The daily doses were 40 mg for afatinib and 250 mg for gefitinib. Although overall survival data had not yet matured, afatinib showed a longer treatment duration before failure compared to gefitinib (13.7 months versus 11.5 months). This difference is attributed to afatinib’s irreversible binding to ATP at the EGFR, which allows it to remain bound for a longer period compared to gefitinib [58]. Afatinib received FDA approval as an irreversible TKI in 2013 and is marketed under the name Gilotrif^®^ [59,60].

However, second-generation TKIs were unable to overcome the T790M mutation, and additional mutations were identified within exons 18–21, including D761Y, L747S, G719X, S768I, L861Q, and T854A [61]. Resistance to TKIs was also observed in NSCLC due to histologic transformations associated with mesenchymal transition, which led to EGFR amplification and increased levels of P13K and MAPK. This, in turn, resulted in enhanced cancer cell proliferation [61].

### 3.3. Third-Generation TKI

**Figure A4:**
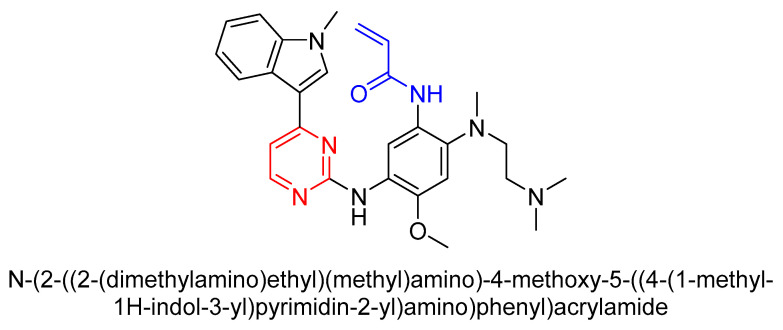
Chemical structure of osimertinib. Red, pyrimidine-based core; blue, covalent warhead.

A drug was needed to address the T790M mutation in NSCLC, a challenge that first- and second-generation TKIs could not overcome, while also maintaining the irreversible binding properties seen in second-generation TKIs. This need led to the development of a third-generation TKI. The first of these, initially named “WZ4002,” was later renamed osimertinib [62]. Unlike the anilinoquinazoline-based first- and second-generation TKIs, osimertinib is pyrimidine-based (Figure A4) [63].

**Figure 6:**
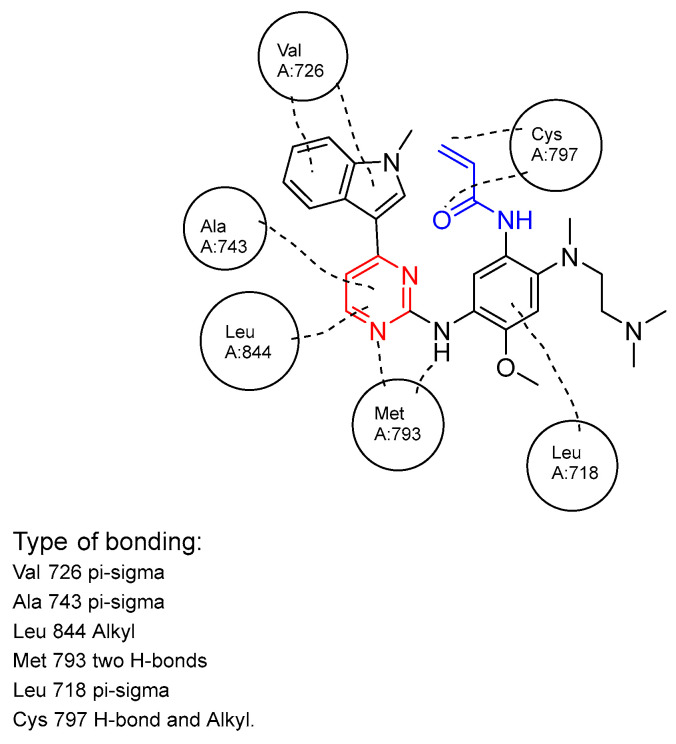
Schematic representation of osimertinib bound to the active site of EGFR tyrosine kinase, with each amino acid involved and their interaction type [43].

Osimertinib, along with other third-generation TKIs, can effectively overcome the T790M mutation, leading to increased potency. This effectiveness is due to the combination of a covalent warhead and a pyrimidine core [56], which enables stronger binding and irreversible inhibition of EGFR kinase activity. Specifically, a covalent bond forms between the warhead and the C797 residue, while two hydrogen bonds form with the Met793 and Ser797 amino acids (Figure 6) [64]. This enhanced binding increases osimertinib’s potency compared to earlier generations. This is evidenced by osimertinib’s lower IC50 values against T790M (0.10–4.00 nM) compared to gefitinib (320–510 nM) and afatinib (141–196 nM) [65], demonstrating that third-generation TKIs are effective at lower concentrations in vitro due to their ability to bind to T790M.

From December 2014 to March 2016, AstraZeneca funded Phase III trials to compare osimertinib and gefitinib as first-line treatments for patients with the T790M mutation in NSCLC [66]. In these trials, 279 patients received oral osimertinib tablets (80 mg once daily), while 277 patients were treated with oral gefitinib tablets (150 mg once daily). The results showed that the median overall survival was 38.6 months for the osimertinib group and 31.8 months for the gefitinib group [66]. After three years, 28% of patients in the osimertinib group and 9% in the gefitinib group were still receiving treatment from the trial. Both treatment groups experienced adverse effects, with 98% of patients encountering at least one. Severe grade 3 adverse effects, which impact daily activities, occurred in 42% of the osimertinib group (3% of which were fatal) and 47% of the gefitinib group (4% of which were fatal) [66]. The most common adverse effects in both groups were diarrhea and rash (Table 5).

This trial involved a larger patient population and included a broader age range, including individuals over 75 years old (Table 6). It also examined treatment effects in both smokers and non-smokers, as well as Asians and non-Asians, addressing gaps present in earlier clinical trials (Table 2).

Trial results demonstrated that osimertinib was more effective than the first-generation TKI gefitinib for treating NSCLC with the T790M mutation and offered a more manageable side effect profile. This progression illustrates the successful advancement of TKIs from traditional chemotherapy agents, such as cisplatin and carboplatin, to first-generation TKIs like gefitinib, and then to third-generation TKIs like osimertinib. These advancements have enabled the effective targeting of the T790M mutation while ensuring irreversible action. Over the course of these trials, there has been a notable increase in TKI efficacy along with a reduction in toxicity. Fewer adverse effects enhance patients’ overall quality of life—physically, mentally, and socially—during anti-cancer treatment. The convenience of oral tablet administration, as opposed to regular IV treatments, improves patient adherence and spares them from disrupting personal or work life [24].

Osimertinib was approved by the FDA as an anti-cancer treatment in 2015 [67], and by the European Union (EU) in 2016. It is marketed under the name Tagrisso^®^ [68].

## 4. Future for TKIs

Despite the successes of third-generation TKIs in overcoming T790M mutations, recent research from Yonsei University’s Department of Medicine in Seoul, South Korea, has revealed that all three generations of TKIs have been ineffective against NSCLC patients harboring the C797S mutation in the EGFR [69]. This mutation, which arises as a resistance mechanism to osimertinib, involves a substitution of cysteine with serine in the ATP-binding site, thereby preventing the formation of a covalent bond between the mutant EGFR and osimertinib. The C797S mutation accounts for 10–26% of osimertinib-resistant cases [69]. This underscores the need for a new generation of TKIs specifically targeting EGFR C797S-mutated NSCLC.

**Figure A5:**
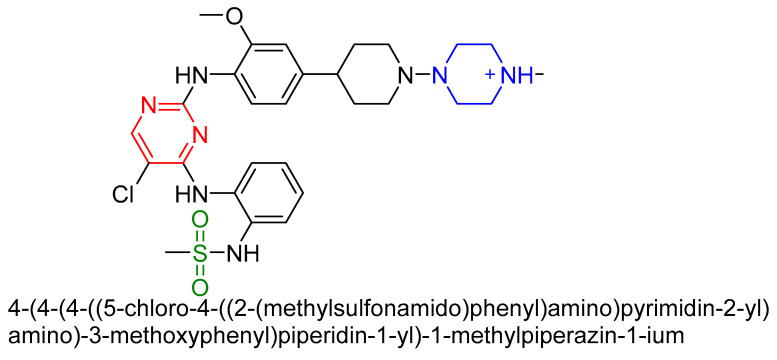
Chemical structure of BBT-176. Red, pyrimidine-based core; blue, piperidine ring; green, sulfone.

In response to this challenge, Yonsei University has developed a reversible TKI known as BBT-176. BBT-176 is a potent inhibitor of both the C797S and T790M mutations [70]. It was designed as an irreversible ATP-competitive inhibitor to address the resistance and reduced efficacy of osimertinib against the C797S mutation. BBT-176 features a pyrimidine ring structure similar to osimertinib (Figure A5) [69].

The structure of BBT-176 allows for two hydrogen bonds with Met793: one from the top nitrogen of the pyrimidine ring and another from the secondary amine linking the pyrimidine to the aromatic chain. Additionally, the piperidine ring forms salt bridges with Glu904 and Asp800. Notably, differences in the spatial positions of Lys745, Asp855, and Gly857 are observed between the active and inactive kinase domains [69]. The interaction between the sulfone group on BBT-176 and Lys745 could enhance its potency against the C797S-mutated EGFR. BBT-176 shows an average IC50 of 3.83 nM for the C797S EGFR, compared to osimertinib’s average IC50 of 300.88 nM [69]. This indicates that BBT-176 is more effective in vitro against the C797S mutation, justifying further investigation through phase trials.

Currently, Phase I human trials are underway at Yonsei University to establish a baseline understanding of BBT-176 in vivo and to determine the optimal dose for Phase II trials [71]. In March 2022, a trial was conducted involving five individuals with the C797S mutation who received BBT-176 daily for six weeks. The trial aimed to assess efficacy by measuring tumor size reduction (Table 7), with treatment continuing until disease progression or patient intolerance to BBT-176 occurred. The drug was administered orally once daily at doses ranging from 20 mg to 600 mg [71].

Common adverse effects observed included nausea (*n* = 5), vomiting (*n* = 3), diarrhea (*n* = 3), rash (*n* = 4), pruritus (*n* = 2), and increases in amylase (*n* = 2) and lipase (*n* = 2). Continuous daily dosing of BBT-176 was generally well tolerated with manageable toxicities [71].

Phase I trials support the in vitro findings, demonstrating that BBT-176 effectively inhibits the EGFR with the C797S mutation. This inhibition helps prevent pathogenesis in NSCLC by halting cell growth and proliferation, leading to a reduction in tumor size in the small patient sample. These positive results highlight the potential of BBT-176 and suggest that its effectiveness could be further improved. Future studies will focus on determining the optimal dose and expanding trials to Phase II and III, which will involve a larger number of participants and enable broader data extrapolation.

Zhang and colleagues developed EGFR PROTACs that effectively degrade the EGFRDel19/T790M/C797S mutant [72]. A compound they referred to as 6h reduced EGFR protein levels in Ba/F3 cells, used the Von Hippel-Lindau (VHL)–proteasome pathway, and inhibited downstream signals ERK and AKT [72]. It showed strong growth inhibition with an IC50 of 0.02 μM and is a promising candidate for overcoming C797S mutant resistance [72]. Recently, Zhu and colleagues designed novel PROTACs targeting the C797S mutation, with compound C6 showing potent degradation of EGFRL858R/T790M/C797S and other major EGFR mutants [73]. C6 achieved a DC50 of 10.2 nM and an IC50 of 10.3 nM, working through the ubiquitin–proteasome system [73]. Compound C6 strongly inhibited clonogenicity, arrested the cell cycle in G0/G1, promoted apoptosis, and blocked EGFR downstream signaling in an H1975-TM xenograft model, presenting a promising approach to overcoming osimertinib resistance [73].

Wang and coworkers identified a compound (and named it 11eg) as a potent and selective EGFR C797S inhibitor with an IC50 of 0.053 μM [74]. It effectively targets EGFRL858R/T790M/C797S, induces cell cycle arrest and apoptosis, and shows strong anticancer activity in vitro and in vivo [74]. 11eg is selective against mutant EGFRs and exhibits a tumor growth inhibition of 70.6% in xenograft models with minimal toxicity, making it a promising candidate for treating NSCLC with EGFR C797S mutations [74].

Kageji and colleagues discovered a 2,4-diaminonicotinamide derivative (and named it 5j) as a potent and selective inhibitor of EGFRs with triple mutations (del19/T790M/C797S and L858R/T790M/C797S), while sparing the wild-type EGFR [75]. They confirmed by co-crystal structure analysis of j5 with EGFR del19/T790M/C797S, that it improves potency through new hydrogen bond formations [75].

Lee and colleagues reported BI-4732 as a novel fourth-generation EGFR-TKI [76]. They found that BI-4732 is a potent EGFR inhibitor effective against EGFR_C797S-mediated osimertinib resistance and EGFR-activating mutations [76]. It showed synergistic effects with osimertinib at lower doses and strong antitumor activity in an intracranial model, with good blood–brain barrier penetration. These results support BI-4732’s potential for clinical development as a selective EGFR-TKI targeting a broad range of mutations [76].

For patients with NSCLC resistant to EGFR-TKIs, combining immunotherapy with chemotherapy and/or anti-angiogenesis is a promising strategy. Those with specific conditions—such as uncommon EGFR mutations, the EGFR L858R mutation, PD-L1 ≥ 50%, prior use of anti-angiogenic drugs, and a negative T790M mutation—might benefit more from immunotherapy after failing EGFR-TKI treatment [77].

Li and colleagues conducted a study of 237 advanced NSCLC patients with EGFR mutations: 160 received immunotherapy in various combinations: ICI alone, ICI plus anti-angiogenesis, ICI plus chemotherapy, or ICI plus both anti-angiogenesis and chemotherapy [78]. Seventy-seven received standard chemotherapy, with or without anti-angiogenesis. Patients who received a combination of immunotherapy, anti-angiogenesis, and chemotherapy after failing EGFR-TKIs had better overall response rates, disease control rates, and median progression-free survival [78].

A study conducted by Yu et al. assessed the effect of combining anlotinib with immune checkpoint inhibitors (ICIs) in NSCLC patients resistant to EGFR-TKI [79]. Compared to those receiving platinum-pemetrexed chemotherapy, patients on the anlotinib–ICI combo showed improved overall survival (OS), irrespective of PD-L1 levels, suggesting the combination’s benefits are independent of PD-L1 expression [79].

Zhou and colleagues studied the efficacy and safety of PD-1 inhibitors in 102 EGFR-mutant NSCLC patients who had failed EGFR-TKIs and received multiple lines of immunotherapy [80]. The study found that PD-1 inhibitors improved survival, particularly in the EGFRL858R and EGFRT790M-negative subgroups, with a trend towards better outcomes when combined with other therapies. Toxicity was well tolerated. Their real-world study supported findings from clinical trials and expanded the patient population to confirm similar survival benefits [80].

Epigenetic inhibitors’ activity is often disrupted in various cancers, including CML. However, when combined with TKIs, they may present promising therapeutic strategies for treating CML cells. For a detailed discussion on the epigenetic pathways involved in CML pathogenesis and tumor cell resistance to TKIs, readers are encouraged to refer to [81].

Coffin–Lowry syndrome (CLS) is a rare X-linked neurodevelopmental disorder that impacts chromatin remodeling, leading to structural abnormalities in male patients, often accompanied by significant neurodevelopmental challenges. The molecular basis of CLS lies in loss-of-function mutations in the RSK2 gene (also known as RPS6KA3), which encodes a serine/threonine protein kinase crucial for chromatin design and cellular signaling pathways in humans [82].

Therefore, TKIs play a crucial role in the treatment of certain cancers, including chronic myeloid leukemia (CML), by targeting and inhibiting specific enzymes involved in cancer cell growth. In CML, TKIs are particularly effective in blocking the breakpoint cluster region (BCR) and Abelson (ABL) gene fusion TK, which is a key driver of the disease. The combination of TKIs with other therapeutic strategies, such as epigenetic inhibitors, offers potential in overcoming resistance and enhancing treatment efficacy.

## 5. Conclusions

This review highlights the evolution of TKIs as targeted therapies for cancer, particularly NSCLC, from traditional chemotherapy treatments. The progression reveals the limitations of traditional treatments such as cisplatin, characterized by high non-selectivity and adverse effects observed during trials, which significantly impact patients’ quality of life. Subsequent advancements led to the development of first-generation TKIs, particularly gefitinib, based on anilinoquinazoline, which demonstrated improved efficacy compared to traditional chemotherapy through Phase III trials. However, gefitinib’s limitation was its inability to overcome the T790M mutation, a challenge also faced by second-generation TKIs like afatinib, despite the addition of a covalent warhead. The development of third-generation TKIs such as osimertinib has proven effective in overcoming the T790M mutation. This is achieved by combining a pyrimidine-based core with a covalent warhead, enabling irreversible binding to the EGFR. This efficacy has been demonstrated in both in vitro and in vivo trials. However, recent research conducted in South Korea has revealed that third-generation TKIs are ineffective in treating NSCLC with the C797S mutation. As a result, trials of the molecule BBT-176 have commenced, showing promising results in vitro and in vivo for anti-cancer treatment targeting this mutation. Several groups have developed promising drugs to overcome the C797S mutation by targeting and degrading the mutant EGFR. These drugs induce cell cycle arrest and apoptosis in affected cells. However, research is ongoing to fully understand their efficacy and optimize their use. Combining different generations of TKIs, such as BI-4732 and osimertinib, can enhance antitumor activity, demonstrating a potent synergistic effect [82].

Combining different generations of TKIs, such as BI-4732 and osimertinib, can enhance antitumor activity, demonstrating a potent synergistic effect [76]. Furthermore, combining immunotherapy with chemotherapy and/or antiangiogenesis is a promising strategy for patients who have failed EGFR-TKI treatment, potentially offering greater benefits.

Cancer research is dynamic and ever-evolving, as evidenced in this review. The evolution of TKIs demonstrates their adaptability to keep pace with advancements in the field. Researchers continually explore structural modifications to enhance their binding affinity to the EGFR and effectively target its various mutations. Additionally, TKIs have progressively improved in terms of ease of administration and reduced adverse effects with each generational advancement, making them more patient friendly.

From a broader perspective, the successful development of TKIs represents a significant stride forward in the treatment of cancer. This offers hope that ongoing research efforts and increased funding will lead to the discovery of additional therapies with enhanced efficacy and further improvements in the quality of life for patients grappling with this challenging diagnosis. Combining immunotherapy with chemotherapy and/or anti-angiogenesis is a promising strategy for patients who have failed EGFR-TKI treatment, potentially offering greater benefits.

The combined paradigm in cancer treatment is increasingly recognized as a preferred strategy because it targets cancer cells at multiple stages and through various mechanisms. This approach typically enhances overall efficacy compared to single-agent therapies by addressing the complexity of cancer more comprehensively and reducing the likelihood of resistance. Evidence supporting this strategy includes the successful combination of different generations of TKIs [76], the integration of immunotherapy with chemotherapy and/or antiangiogenesis agents [77,78], and other multi-faceted approaches.

## Figures and Tables

**Table 1:** Approved TKIs to treat NSCLC.

Name	Mode of Action	Therapeutic Target	Administration in Adults	Approval Year	Reference Number
First-generation TKI					
Gefitinib [27]	Reversible inhibitor for EGFR	Advanced/ Metastatic NSCLC with exon 19 and 21 mutations	Oral Tablets	2002 Japan 2003 FDA	NDA 206995
Erlotinib [28]	Reversible inhibitor for EGFR	Advanced/ Metastatic NSCLC	Oral Tablets	2004 FDA	NDA 021743
Icotinib [29]	Inhibits MAPK/ERK and AKT in EGFR	Advanced/ Metastatic NSCLC, even exon 19 and 21 mutations	Oral Tablets	2012 China	220224-91-5
Second-generation TKI					
Afatinib [30]	Irreversible inhibitors for EGFRs HER2 and HER4	Exon 19 or exon L8585 in NSCLC	Oral Tablets	2013 FDA 2013 EMA	NDA 201292 EMEA/H/C/002280
Brigatinib [31]	Selective inhibitor of ALK, including T790M	ALK-positive metastatic NSCLC	Oral Tablets	2017 FDA 2018 EMA	NDA 208772 EMEA/H/C/004257
Dacomitinib [25]	Irreversible inhibitor of EGFR	Metastatic NSCLC, with exon 19 or 21 mutations	Oral Tablets	2018 FDA 2019 EMA	NDA 211288 EMEA/H/C/004094
Capmatinib [32]	Selective inhibitor of MET	Metastatic NSCLC with exon 14 mutation	Oral Tablets	2020 FDA	NDA 213383
Third-generation TKI					
Osimertinib [33]	Inhibits EGFR with exon 19 and T790M mutations	Resistant NSCLC from T790M mutation	Oral Tablets	2015 FDA 2016 EMA	NDA 208065 EMEA/H/C/003905
Olmutinib [34]	Inhibits EGFR with T790M mutation	Resistant NSCLC from T790M mutation	Oral Tablets	2016 South Korea	HM61713
Lorlatinib [35]	Inhibits ALK and ROS1	ALK-positive metastatic NSCLC	Oral Tablets	2018 FDA 2019 EMA	NDA 210868 EMEA/HC/004664
Almonertinib [36]	Inhibits EGFR, including T790M mutations	Advanced/Metastatic NSCLC, with T790M mutation	Oral Tablet	2020 China	HS-10296
Mobocertinib [37]	Inhibits EGFR with exon 20 mutations	Resistant NSCLC from exon 20 mutation	Oral Tablets	2021 FDA	NDA 214164

AKT, protein kinase B; ALK, anaplastic lymphoma kinase; ARK, extracellular signal-regulated kinase; HER2, human epidermal growth factor receptor 2; HER4, human epidermal growth factor receptor 4; MAPK, Mitogen-activated protein kinase; MET, Met tyrosine kinase

**Table 2:** Summary of gefitinib against carboplatin trials. Includes response rates and toxicity report [48].

Characteristic		Gefitinib Group	Carboplatin Group
		No. of Patients (% of Patients)	
Response	Complete	5 (4.4)	0
	Partial	79 (69.3)	35 (30.7)
Stabilized NSCLC		18 (15.8)	56 (49.1)
Progressive disease		11 (9.6)	16 (14.0)
Grade 1 side effect	Diarrhea	32	7
	Appetite Loss	7	39
	Rash	38	8
	Neuropathy (sensory)	0	28
	ALT elevation	20	31
	Anemia	19	35
	Thrombocytopenia	8	25
Progression free	Date	Survival (PFS)	
	May 2009	10.4 months	5.5 months
	December 2009	10.8 months	5.4 months

**Table 5:** Osimertinib against gefitinib trials. Includes survival rates and toxicity report for any grade of adverse effect [66].

Characteristic		Osimertinib Group (N = 279)	Gefitinib Group (N = 277)
		No. of Patients (% of Patients)	
Overall survival	At 12 months	89	83
	At 24 months	74	59
	At 36 months	54	44
Any grade of adverse effects	Diarrhea	167 (60)	162 (58)
	Rash/Acne	164 (59)	219 (79)
	Nail Effects	108 (39)	95 (34)
	Dry Skin	106 (38)	102 (37)
	Stomatitis	82 (29)	60 (22)
	Decreased Appetite	66 (24)	58 (21)
	Cough	60 (22)	50 (18)
	Nausea	55 (20)	55 (20)
	Constipation	51 (18)	39 (14)
	Anemia	44 (16)	27 (10)

**Table 6:** Subgroup review for osimertinib against gefitinib trials. Total of 556 patients were recruited [66].

Subgroup		No. of Patients
Sex	Male	206
	Female	350
Age	Under 65	298
	Over 65	258
Race	Asian	347
	Non-Asian	209
Smoking history	Yes	199
	No	357

**Table 7:** Phase I trial results from Yonsei University for BBT-176 efficacy against C797S mutation in NSCLC [71].

Dose (mg)	Age	Gender	Race	Tumor Size Change (%)
160 to 320	43	Female	Asian	−30.3
320	39	Male	Asian	0.0
320	52	Female	Asian	−12.1
480	67	Female	Asian	−11.8
480	54	Male	Asian	−26.3

## Data Availability

Data sharing is not applicable.
